# Clinical features and imaging examination assessment of cervical lymph nodes for thyroid carcinoma

**DOI:** 10.1186/s12885-023-11721-5

**Published:** 2023-12-12

**Authors:** Bei Wei, Jincao Yao, Chanjuan Peng, Shanshan Zhao, Hui Wang, Liping Wang, Xi Zhu, Yuting Kong, Liyu Chen, Dong Xu

**Affiliations:** 1 Department of Ultrasound, Zhejiang Cancer Hospital, Hangzhou Institute of Medicine (HIM), Chinese Academy of Sciences, No.1 East Banshan Road, Gongshu District, Hangzhou, 310022 Zhejiang Province China; 2 Chinese Academy of Sciences, Key Laboratory of Head & Neck Cancer Translational Research of Zhejiang Province, No.1 East Banshan Road, Gongshu District, Hangzhou, 310022 Zhejiang Province China; 3 Zhejiang Provincial Research Center for Cancer Intelligent Diagnosis and Molecular Technology, No.1 East Banshan Road, Gongshu District, Hangzhou, 310022 Zhejiang Province China

**Keywords:** Thyroid carcinoma, Cervical lymph node Metastasis, Clinical characteristics, Ultrasound, CT

## Abstract

**Backgrounds:**

The purpose of this study is to investigate the relationship between clinical characteristics and cervical lymph node metastasis (LNM) in patients with thyroid carcinoma, as well as estimate the preoperative diagnosis values of ultrasound (US) and contrast enhanced computed tomography (CECT) examinations on the neck for detection of cervical LNM in thyroid carcinoma.

**Methods:**

A retrospective analysis of 3 026 patients with surgically proven thyroid carcinoma was conducted. Patients’ clinical characteristics, including gender, age, tumor size, bilateral lesions, multifocality, adenomatous nodules, Hashimoto’s thyroiditis (HT), and extrathyroidal extension, were collected to explore their association with cervical LNM in thyroid carcinoma. Preoperative assessments for central lymph node metastasis (CLNM) and lateral lymph node metastasis (LLNM) were conducted through US and CECT. The diagnostic value of US, CECT and US combined with CECT for detection of LNM located in various cervical compartments was estimated based on the pathological results.

**Results:**

The risk of cervical LNM was higher in thyroid cancer patients who were male, age < 55 years old, tumor size > 10 mm, bilateral lesions, and extrathyroidal extension, while multifocality, adenomatous nodules and HT had no significant effect on LNM. US, CECT and US combined with CECT all had a higher sensitivity to LLNM (93.1%, 57.8%, 95.4%) than to CLNM (32.3%, 29.0%, 43.4%). US and CECT had a high specificity to both CLNM and LLNM (94.3–97.8%).

**Conclusion:**

Preoperative clinical characteristics and imaging examinations on patients with thyroid carcinoma are crucial to the evaluation of cervical lymph nodes and conducive to individualizing surgical treatments by clinicians. US combined with CECT are superior to single US or CECT alone in detection of CLNM and LLNM.

## Introduction

Thyroid carcinoma is the most common endocrine tumor and its incidence rate has been increasing in recent years, making it the fourth leading cancer worldwide [1]. It includes four pathological types: papillary thyroid carcinoma (PTC), follicular thyroid carcinoma (FTC), anaplastic thyroid carcinoma (ATC) and medullary thyroid cancer (MTC). Among them, PTC is the most prevalent type, overall survival at 14 years was 82% for PTC without lymph node metastases and 79% with nodal metastases (*p* < 0.05). [2, 3]. Currently, surgery remains the main treatment for thyroid carcinoma, and the accurate preoperative evaluation of lymph node metastasis (LNM) is conducive to developing a suitable surgical plan, reducing the local recurrence rate and avoiding complications from reoperations [4, 5]. Cervical LNM in thyroid carcinoma is classified into two types: central lymph node metastasis (CLNM) and lateral lymph node metastasis (LLNM). According to the lymphatic drainage pathway, LNM typically occurs initially in the central compartment before involving the lateral compartment, sometimes presenting as skip metastasis [6, 7]. The incidence of CLNM in PTC patients is high, ranging from 21 to 60% [8, 9], and approximately 20% of PTC cases are accompanied by LLNM at the initial diagnosis [10, 11]. Considerable studies have shown that LLNM might increase the risk of local recurrence and reduce the survival rate [10]. High-resolution ultrasound is the preferred imaging method for preoperative evaluation of cervical lymph nodes by clinicians [12]. Suspicious lymph nodes with a short diameter ≥ 8–10 mm are often diagnosed as malignant through ultrasound-guided fine-needle aspiration (FNA) [13]. However, only 38–59% of these lymph nodes can be detected by preoperative ultrasound examination due to the presence of incompletely imaged deep anatomical structures covered by the thyroid gland, as well as structures covered by bones or gas [14], Additionally, only about half of the lymph nodes found intraoperatively are detected by preoperative ultrasound. According to *American Thyroid Association Management Guidelines*, contrast enhanced computed tomography (CECT) provides a supporting examination on patients in clinically suspected disease progression. Computed tomography (CT) is less dependent on the operator and more effective in describing airway obstruction or periesophageal lymph nodes. However, there are limited studies worldwide on the preoperative evaluation of combined ultrasound and CECT in the detection of cervical LNM in thyroid carcinoma, and the existing results are contradictory [15, 16]. This study aims to achieve a comprehensive understanding of the importance of US and CECT in the preoperative evaluation of cervical LNM in thyroid carcinoma, improve the preoperative diagnosis of LNM, and ultimately assist clinicians to individualize lymph node dissection planning. Integrating the clinical characteristics of patients with thyroid carcinoma, the study analyzed the diagnostic value of US, CECT and US combined with CECT for detection of cervical LNM in thyroid carcinoma.

## Methods

### Subjects

**Fig. 1:**
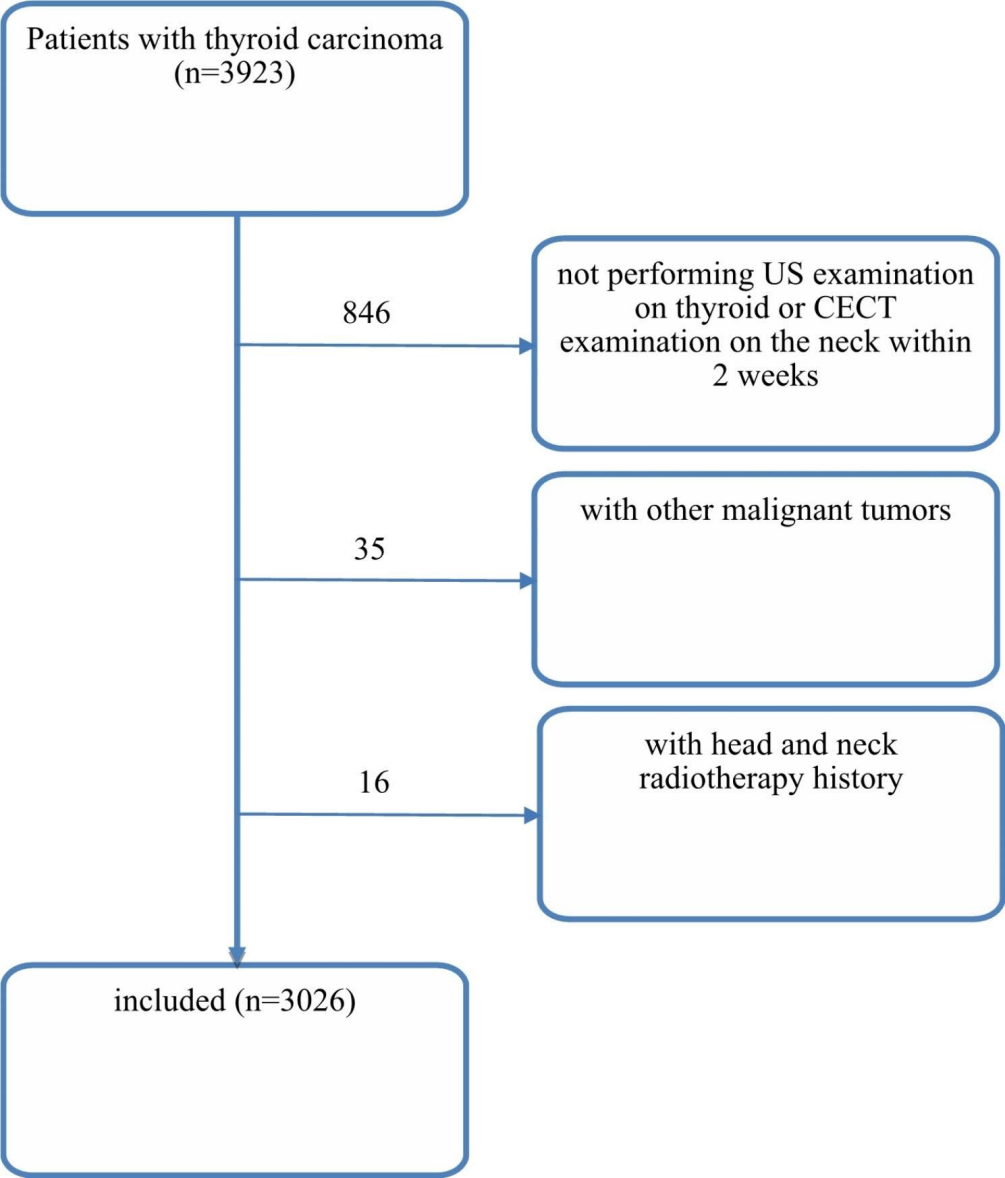
Patients-screening flowchart

This study retrospectively analyzed 3 923 patients with thyroid carcinoma treated in Zhejiang Cancer Hospital from January 2018 to June 2021. It was approved by the Ethics Committee of Zhejiang Cancer Hospital (IRB-2020-287) and conducted in accordance with the ethical standards of the 1964 Helsinki Declaration. The need for informed consent from all patients was waived due to the Ethics Committee of Zhejiang Cancer Hospital. The inclusion criteria are as follows: (1) undergoing surgery; (2) confirmation of thyroid carcinoma by postoperative histopathology; (3) US examination on thyroid and CECT examination on the neck within 2 weeks before surgery. The exclusion criteria are: (1) with head and neck radiotherapy history; (2) with other malignant tumors not involved in this investigation; (3) obvious abnormal liver and kidney function; (4) low compliance. Based on these criteria, 3 026 patients with thyroid carcinoma were enrolled in this study, among whom 763 were males (with mean age of 45 ± 13 years) and 2 263 were females (with mean age of 46 ± 12 years). They all underwent thyroidectomy and central cervical lymph node dissection and their thyroid carcinoma was confirmed by postoperative pathology, among whom 2 962 were diagnosed with PTC, 28 with FTC, 3 with ATC and 33 with MTC. Besides, under China’s *Guidelines for diagnosis and treatment of thyroid nodules and differentiated thyroid carcinoma* [17], all the patients in this study underwent central lymph node dissection and pathologically confirmed lateral cervical lymph node dissection, except the ATC patients, the patients with unilateral thyroid carcinoma underwent lobectomy + isthmus resection + ipsilateral central lymph node dissection, the patients with bilateral thyroid carcinoma underwent total thyroidectomy + bilateral central lymph node dissection, and the patients with LLNMs that were suspected by preoperative US or CT underwent lateral cervical lymph node dissection after confirmed by fine needle aspiration biopsy or intraoperative biopsy, the whole participants-screening process of which is shown in Fig. 1. Moreover, among them 2 575 underwent simple central lymph node dissection, 382 did central + unilateral lymph node dissection, and 69 did central + bilateral lymph node dissection, A total of 2575 patients were confirmed with the true negative LLNM through imaging review conducted three months after surgery. Accordingly, the patients were classified into LNM group (n = 1226, 40.52%) and non-LNM group (n = 1800, 59.48%), with regarding to Hashimoto’s thyroiditis (HT), the presence of Hurthle cells and lymphocyte inflammation with germinal centers in thyroid parenchyma and stroma was defined as the main pathological diagnostic criterion [18, 19].

### Ultrasonography and image analysis

Ultrasound examinations were performed in all the 3 026 cases. Philips iU 22 and GG Logiq E9 color Doppler ultrasonic diagnostic instruments with 9 L-4 linear array probe and the frequency of 10 ~ 12 MHz were used. The patients were told to take the supine position, and routine US examination was performed on their thyroid and cervical lymph nodes. A double-blind method was adopted to independently analyze the pre-stored images by two attending radiologists with more than 5 years’ experience in thyroid US. When the results were conflicting with each other, a closer analysis of the corresponding image would be requested from a superior radiologist. Cervical LNM would be recognized as positive when the ultrasonic image analytical results of cervical lymph nodes met the specific criteria: round-shaped, focal or diffuse hyperechogenicity, microcystis, microcalcification and loss of central hilar echogenicity [20, 21].

### CT examination and image analysis

A multi-slice CT scanner (Siemens 40) or a 64 multidetector scanner (LightSpeed VCT; GE Healthcare, Waukesha, WI, USA) with tube voltage of 120 kV, tube current of 150 mA, section thickness of 3 mm and section interval of 3 mm was used. Contrast-enhanced scans were performed after an intravenous injection of 80–100 ml of nonionic iodinated iopamidol containing 370 mg iodine per ml (Isovue 370, Bracco Healthcare, Princeton, NJ) at 3–4 ml/s. Arterial and venous phase images were obtained at 25 and 60 s, respectively. The pre-stored images were independently analyzed using double-blind method by two attending radiologists who were with more than 5 years’ experience and unaware of the ultrasonic and surgical pathological results. If their results were inconsistent, a closer analysis of the corresponding image would be requested from a superior radiologist. Cervical LNM would be recognized as positive if the CECT image analytical results of the cervical lymph nodes met the specific criteria: calcification, cystic or necrotic changes, heterogeneous enhancement, and significant enhancement without hilar vessel enhancement [22].

### Statistical analysis

Statistical analyses were performed with SPSS version 25.0 software (IBM Corporation, Armonk, NY, USA). *P* value was selected as < 0.05, indicating a statistically significant difference, and the pathological diagnosis of surgical specimens was set as the gold standard. The consistency between US and CECT examinations on the 3 026 cases was evaluated by kappa coefficient. The diagnostic value of US and CECT in detection of LNM was determined to include sensitivity, specificity, positive predictive value (PPV), negative predictive value (NPV) and diagnostic accuracy. McNemar test was adopted to determine whether there was a significant difference in the sensitivity and specificity among US, CECT and US combined with CECT. Statistical significance of difference between clinical characteristics was determined by χ^2^ test, the clinical characteristics with *P* < 0.05 were used to investigate the factors for LNM, and the relationship between clinical characteristics and LNM was analyzed by multivariable analysis.

## Results

### Clinical characteristics and cervical LNM

Among all the 3 026 patients, 1 987 underwent lobectomy plus isthmus resection and 1 039 underwent total or near total thyroidectomy. Moreover, based on the preoperative imaging findings, all the patients underwent preventive or therapeutic central lymph node dissection. Among all them again, 1 171 had metastatic lymph nodes in the central cervical compartment, and among the 451 patients undergoing lateral cervical lymph node dissection, 389 had metastatic lymph nodes in the lateral cervical compartment. Table 1 demonstrates the statistical significance of the relationship between cervical LNM and clinical characteristics.

**Table 1:** Relationship between clinical characteristics and cervical LNM.

	LNM (n = 1226)	Non-LMN (n = 1800)	*P* value	OR(95% confidence interval) *P*-value
Gender				
Male	379(49.7%)	384(50.3%)	0.000	0.596(0.497–0.715),0.000
Female	847(37.4%)	1416(62.6%)		
Age ≥ 55				
No	993(44.2%)	1253(55.8%)	0.000	0.495(0.408–0.601),0.000
Yes	233(29.9%)	547(70.1%)		
Tumor size				
≤ 10 mm	591(28.9%)	1453(71.1%)	0.000	3.534(2.963–4.215),0.000
>10 mm	635(64.7%)	347(35.3%)		
bilateral lesions				
No	877(36.1%)	1553(63.9%)	0.000	1.576(1.165–2.133),0.003
Yes	349(58.6%)	247(41.4%)		
Multifocality				
No	751(35.4%)	1371(64.6%)	0.000	1.251(0.963–1.625),0.093
Yes	475(52.7%)	427(47.3%)		
Adenomatous nodules				
No	990(41.7%)	1385(58.3%)	0.012	0.833(0.682–1.017),0.073
Yes	236(36.3%)	415(63.7%)		
HT				
No	977(40.3%)	1445(59.7%)	0.691	
Yes	249(41.2%)	355(58.8%)		
The presence/absence of extrathyroidal extension				
Intrathyroidal	389(41.5%)	981(58.5%)	0.000	1.650(1.437–1.894),0.000
Capsule invasion	697(48.3%)	745(51.7%)		
Extrathyroidal extension	140(65.4%)	74(34.6%)		

OR odds ratio for relative risk associated with each feature

Bilateral lesions, bilateral thyroid involvement; Multifocality, the number of lesions no less than 2; Intrathyroidal, tumor limited to the thyroid; Capsule invasion, the tumor invading the capsule; Extrathyroidal extension, the tumor invading strap muscles, subcutaneous soft tissues, larynx, trachea, esophagus, or recurrent laryngeal nerve

According to this table, cervical LNM rate of thyroid carcinoma is associated with patients’ gender, age, tumor size, bilateral lesions, multifocality, adenomatous nodules and extrathyroidal extension. Furthermore, the risk of cervical LNM was higher in thyroid cancer patients who were male, age < 55 years old, tumor size > 10 mm, bilateral lesions, and extrathyroidal extension. The difference between cervical LNM and each of the factors was statistically significant (*P* < 0.05), except for multifocality, adenomatous nodules and HT.

**Table 2:** Consistency of the diagnosis of LNM in different cervical compartments by US and CECT.

Cervical compartment(s)			CECT		kappa
			+	-	
The entire(n = 3026)	US	+	432	320	0.576
		-	116	2158	
The central(n = 3026)	US	+	253	230	0.486
		-	164	2379	
The lateral (n = 3026)	US	+	254	231	0.616
		-	29	2512	

### Diagnostic value of imaging in cervical LNM of thyroid carcinoma

Table 2 shows the consistency of the diagnosis of cervical LNM in different cervical compartments by US and CECT. The two types of imaging examinations are moderately consistent in identifying lymph node invasion (kappa: 0.4–0.75).

Table 3 shows the diagnostic results of US, CECT and US combined with CECT in detection of cervical LNM from the perspectives of the sensitivity, specificity, PPV, NPV and accuracy, and Table 4 reveals the statistical results in the sensitivity and specificity of the metastasis detection by different imaging examinations. In terms of the sensitivity, in the central cervical compartment, US and CECT were both less than 40%, respectively 32.3% and 29.0%, while US combined with CECT increased significantly (*P* < 0.05), compared with single US. In the lateral compartment, US, CECT and US combined with CECT examinations were 93.1%, 57.8% and 95.4%, respectively. Here should be noted that US combined with CECT was significantly higher than US (*P* < 0.05). In the entire cervical compartments, US combined with CECT was 56.4%, significantly higher than US (*P* < 0.05). In each cervical compartment, US was higher than CECT, although the increasing of sensitivity with a reduction of specificity, which was still higher than 90%. Nevertheless, in terms of the specificity, US, CECT and US combined with CECT were comparable both in the central compartment (94.3%, 95.8% and 92.5% respectively) and in the lateral compartment (95.3%, 97.8% and 94.6% respectively). CECT, US and US combined with CECT ranked from the highest to the lowest, no matter in the entire, central or lateral compartment, the significances among which were all statistically significant (*P* < 0.05). With respect to the diagnostic accuracy, US, CECT and US combined CECT showed 70.3%, 70.0% and 73.5% in the central cervical compartment, 95.0%, 92.7% and 94.7% in its lateral compartment, and 75.0%, 71.4% and 76.5% in the entire cervical compartments, correspondingly.

**Table 3:** Diagnostic results of cervical LNM by US, CECT and US combined with CECT.

Cervical compartment(s)			Pathological diagnosis		Sensitivity	Specificity	PPV	NPV	Accuracy
			metastasis	No metastasis					
The entire (n = 3026)	US	+	610	142	49.8%	92.1%	81.1%	72.9%	75.0%
		-	616	1658					
	CECT	+	455	93	37.1%	94.8%	83.0%	68.9%	71.4%
		-	771	1707					
	US combined with CECT	+	692	176	56.4%	90.2%	79.7%	75.3%	76.5%
		-	534	1624					
The central (n = 3026)	US	+	378	105	32.3%	94.3%	78.3%	68.8%	70.3%
		-	793	1750					
	CECT	+	340	77	29.0%	95.8%	81.5%	68.1%	70.0%
		-	831	1778					
	US combined with CECT	+	508	139	43.4%	92.5%	78.5%	72.1%	73.5%
		-	663	1716					
The lateral (n = 3026)	US	+	362	123	93.1%	95.3%	74.6%	98.9%	95.0%
		-	27	2514					
	CECT	+	225	58	57.8%	97.8%	79.5%	94.0%	92.7%
		-	164	2579					
	US combined with CECT	+	371	143	95.4%	94.6%	72.2%	99.3%	94.7%
		-	18	2494					

**Table 4:** Statistical analysis of diagnostic results of cervical LNM by US, CECT and US combined with CECT (*P* value)

Cervical compartment(s)		US VS CECT		US VS US combined with CECT	CECT VS US combined with CECT
The entire	Sensitivity	0.000	0.000		0.000
	Specificity	0.000	0.000		0.000
The central	Sensitivity	0.032	0.000		0.000
	Specificity	0.006	0.000		0.000
The lateral	Sensitivity	0.000	0.004		0.000
	Specificity	0.000	0.000		0.000

## Discussion

The incidence of cervical LNM reaches 40–90% in PTC patients [23], and 2–8% in FTC patients, relatively lower, are more incline to the occurrence of hematogenous metastasis and low-frequency occurrence of LNM [24, 25]. In contrast, MTC patients have a much higher incidence of cervical LNM, reaching 70% [26, 27]. LNM is recognized as an independent risk factor for the high mortality rate of ATC, a rare disease with a very poor prognosis [28]. This study demonstrated that among the 3 026 cases, the incidence of CLNM was 38.7%, and that of LLNM was 15.0%. Cervical LNM is the main risk factor for the high recurrence rate of thyroid carcinoma. If the potential LNM is ignored, the reoperation will be almost inevitable, which might produce the increase both in the difficulty of surgery and in the risk for complications such as recurrent laryngeal nerve injury and parathyroid function decline. Therefore, an accurate preoperative evaluation of LNM is conducive to the preoperative staging of thyroid carcinoma, and has extensive reference for the selection of surgical methods and the prognosis of patients. This study thus conducted a retrospective analysis of 3 026 patients with thyroid carcinoma to investigate the relationship between risk factors for LNM and the clinical characteristics of patients and then provide some reference for the selection of surgical operations for thyroid carcinoma. We found that male, age<55 years, tumor size >10 mm, bilateral lesions, and extrathyroidal extension had a higher risk of cervical LNM, while the multifocality, adenomatous nodules, and HT had no significant effect on LNM. Mao et al. reported that capsule invasion and extrathyroidal extension were the two major risk factors for LNM in PTC, and some other studies showed that HT was not related to LNM in PTC [29, 30], the results of which are both consistent with ours. The high coexistence between HT and PTC has been confirmed by many epidemiological studies, with the range from 20 to 85% [31]. Besides, research showed that the incidence of LNM in patients with PTC accompanied by HT was low [31], which might be due to a significantly larger number of removed lymph nodes from patients with HT and then a lower LNM rate in HT patients. Also, according to related literature, PTC or MTC patients with nodular goiter or follicular adenoma had a lower LNM rate [32]. However, based on our results, thyroid carcinoma patients with adenomatous nodules had a lower risk of CLNM than those without in the central cervical compartment (OR = 0.815, *P* = 0.045), and LLNM had no significant influence on them (OR = 1.015, *P* = 0.924). Based on the high incidence rate of CLNM and the and the limitations of routine preoperative detection methods, clinicians often opt for preventive central lymph node dissection. However, some studies have questioned the necessity of this procedure for low-risk PTC patients, as it does not show long-term improvement in patient prognosis and may increase the risk of complications such as hypocalcemia [33, 34]. Due to our findings that US, CECT and US combined with CECT have a diagnostic sensitivity of less than 45% for detecting CLNM, it is observed that male, age < 55 years old, tumor size >10 mm, bilateral lesions, and extrathyroidal extension had a higher risk of cervical LNM for thyroid cancer patients. As an important risk factor for LLNM, the number of CCLM is increasingly being included in risk stratification [35]. Therefore, we first suggested that patients with thyroid cancer who exhibit the aforementioned characteristics undergo preventive central lymph node dissection. Simultaneously, close attention should be paid to the involvement of lateral neck lymph nodes, which is beneficial for risk assessment and avoiding a second surgery that may affect the quality of life.

Second, to image the neck before thyroid carcinoma surgery is necessary to determine the appropriate scope of surgical resection. Based on the study that between two common preoperative imaging examinations of US and CECT, in the central, lateral and entire cervical compartments, US has a higher sensitivity, while CECT has a higher specificity. US is thus more effective in reducing the rate of missed diagnoses, while CECT helps avoid misdiagnosis. In the entire cervical region, US detected 610 metastases (20%), CECT identified 455 (15%), US combined with CECT revealed 692 cases (22%). The use of US combined with CECT only slightly improved detection compared to US alone, despite being statistically significative. A 2% absolute increase that does not justify radiation exposure in all patients. given the lack of economic analysis and the lack of safety data regarding radiation, contrast-induced acute kidney injury, contrast allergy, among other. Also, for certain pathologic types in PTC, the small size of the lymph node suggests overdiagnosis and upstaging of the disease, a disease that may stable for many years [36]. This study includes almost all types of thyroid cancer, although the sample size for types other than papillary cancer is relatively small. There may be variations in the response to lymph node metastasis among certain subtypes, potentially influencing overall survival. Further research is needed to determine which patient subgroups will benefit effectively from CT. The American Thyroid Association (ATA) guidelines in 2015 indicate that preoperative use of cross-sectional imaging studies (CT, MRI) with intravenous (IV) contrast is recommended as an adjunct to US for patients with clinical suspicion for advanced disease, including invasive primary tumor, or clinically apparent multiple or bulky lymph node involvement, to better characterize tumor invasion and bulky, inferiorly located, or posteriorly located lymph nodes. Preoperative knowledge of these features of the primary tumor or metastases could significantly influence the surgical plan [37]. According to what we discussed earlier that male, age < 55 years old, tumor size >10 mm, bilateral lesions, and extrathyroidal extension had a higher risk of cervical LNM for thyroid cancer patients. Therefore, this study supports the suggestion for preoperative contrast-enhanced computed tomography (CECT) examination for thyroid cancer patients possessing the aforementioned clinical characteristics. For thyroid cancer patients with these characteristics, a preoperative ultrasound combined with CECT examination is suggested. Iodine is generally cleared within 4–8 weeks in most patients, so concern about iodine burden from IV contrast causing a clinically significant delay in subsequent whole-body scans (WBSs) or RAI treatment after the imaging followed by surgery is generally unfounded [38]. By comprehensively evaluating the findings from both modalities, clinicians can make better clinical decisions and select individualized treatment approaches. In this study, ultrasonography and CECT were used to diagnose CLNM, with mean lymph node sizes of 5.4 and 6.7 mm, respectively. The sensitivity to CLNM by US was 32.3%, while that by CECT was lower, which might be owing to the current lack of uniform standard for LNM. Some literatures have reported a higher sensitivity of CECT, which could be attributed to the previous studies’ use of a 5 mm threshold for the central cervical compartment. [16]. However, relying solely on size criteria may not be appropriate for determining CLNM, and the lower sensitivity of CECT in detecting CLNM might be due to the complex anatomical structure at the chest entrance and the relatively small lymph nodes. Although CT is better able to visualize the entire neck region, its ability to interpret imaging results for small lymph nodes is inferior to ultrasound. CT imaging is less sensitive to CLNM than US imaging, which is consistent with the results of some previous studies [39, 40]. Both methods often exhibit lower diagnostic accuracy for CLNM compared to LLNM, consistent with previous studies. [41, 42]. Moreover, a considerable number of studies have shown that LLNM is likely to increase the risk of local recurrence and reduce the survival rate [43], and the recurrence rate of LLNM positive patients is significantly higher than that of CLNM patients [12]. In this study, the sensitivity and specificity of US in the LLNM detection are as high as 93.1% and 95.3%, correspondingly, which might result in a comparatively low rate of missed diagnosis and avoidance of misdiagnosis. Therefore, as the final conclusion, US imaging is finally suggested to be the preferred imaging method for assessing LLNM in patients with thyroid carcinoma.

The current study exposes several limitations. Firstly, due to the technical problems related to lymph node dissection, the lymph nodes evaluated cannot directly correspond with those pathologically examined, while the anatomical area of cervical lymph nodes is specially marked in surgery and imaging data. Second, except for PTC, the incidence rate of the types of thyroid carcinoma is relatively low, only 28 FTC patients, 3 ATC patients and 33 MTC patients enrolled in this study. Third, regardless of the imaging evaluation of LNM, it is necessary to perform lateral cervical dissection for all patients, then obtaining the real sensitivity value in the detection of the lateral neck. The final limitation is that the lateral cervical lymph node dissection was only performed in patients with pathologically proven LNM. For patients who did not undergo LLNM dissection, a negative LLNM result in the early postoperative imaging review is assumed to be a true negative. However, if LLNM is detected during long-term follow-up, it could be due to a false negative result in the preoperative assessment or it may indicate disease recurrence itself.

## Conclusions

Compared to CECT, US demonstrated higher sensitivity in detecting LNM in the central, lateral and entire cervical compartments. The combination of US and CECT was found to be superior to using either modality alone in detecting both CLNM and LLNM. CECT takes a good complement to US in detecting LNM. Based on our finding that the sensitivity of US, CECT and US combined with CECT in the detection of CLNM was all less than 45%, we suggested that patients with thyroid carcinoma having the clinical characteristics as follows: male, age <55 years old, tumor size > 10 mm, bilateral lesions, and extrathyroidal extension, should undergo preventive central lymph node dissection, Additionally, close attention should be paid to the invasion of lateral cervical lymph nodes. These suggestions are beneficial for clinicians in conducting risk assessments and selecting individualized surgical treatments.

## Data Availability

The datasets used and/or analysed during the current study available from the corresponding author on reasonable request.

## Abbreviations

ATC: Anaplastic thyroid carcinoma
CECT: Contrast enhanced computed tomography
CLNM: Central lymph node metastasis
CT: Computed tomography
FTC: Follicular thyroid carcinoma
HT: Hashimoto’s thyroiditis
LLNM: Lateral lymph node metastasis
LNM: Lymph node metastasis
MTC: Medullary thyroid cancer
NPV: Negative predictive value
PPV: Positive predictive value
PTC: Papillary thyroid carcinoma
US: Ultrasound
