# Correction: Clinical features and imaging examination assessment of cervical lymph nodes for thyroid carcinoma

**DOI:** 10.1186/s12885-024-11896-5

**Published:** 2024-01-29

**Authors:** Bei Wei, Jincao Yao, Chanjuan Peng, Shanshan Zhao, Hui Wang, Liping Wang, Xi Zhu, Yuting Kong, Liyu Chen, Dong Xu

**Affiliations:** 1 Department of Ultrasound, Zhejiang Cancer Hospital, Hangzhou Institute of Medicine (HIM), Chinese Academy of Sciences, No.1 East Banshan Road, Gongshu District, 310022 Hangzhou, Zhejiang Province China; 2 Chinese Academy of Sciences, Key Laboratory of Head & Neck Cancer Translational Research of Zhejiang Province, No.1 East Banshan Road, Gongshu District, 310022 Hangzhou, Zhejiang Province China; 3 Zhejiang Provincial Research Center for Cancer Intelligent Diagnosis and Molecular Technology, No.1 East Banshan Road, Gongshu District, 310022 Hangzhou, Zhejiang Province China

**Correction: BMC Cancer 23, 1225 (2023)**

10.1186/s12885-023-11721-5

Following publication of the original article [1], the authors reported an error in the presentation of the affiliations 1 and 2. The affiliations are presented correctly in this correction article.

The original article [1] has been corrected.
